# Sanitary Report of the West Division of the City of Chicago

**Published:** 1864-03

**Authors:** Hiram Wanzer

**Affiliations:** Chicago, Ill.


ARTICLE XII.

### SANITARY REPORT OF THE WEST DIVISION OF THE CITY OF CHICAGO.

BY HIRAM WANZER, M.D., of Chicago, Ill.

Read before the Chicago Medical Society, Friday Evening, Feb. 26, 1864.

MR. PRESIDENT AND GENTLEMEN:—Having been appointed to make a Sanitary report of the West Division of our city, should I wait longer, I should not be better prepared, probably, than at present. The writing a Sanitary Report of the condition of our city, calls for a more extensive experience and more time than I have had to devote to it.

During the last quarter there have been a marked increase in some diseases, especially those classed as zymotic. Small-pox, of the milder form, has abounded more than any other disease of the class. During the month of January last, the deaths from small-pox reported were 55; the month previous 23. Showing how the mortality increases by cold weather, in my opinion the majority of cases were of the milder variety, and have recovered without much medication, never coming under the observation of the physician. I believe the pathology and treatment of small-pox is not well understood by many of our respectable practicing physicians, and can only be learned by practical observation at the bedside. As far as I am acquainted successful vaccination has been a safeguard against the disease in most instances. Chicken-pox has prevailed somewhat extensively, and in some instances has been mistaken for small-pox, which has increased the panic. A few cases of scarlatina and measles have been noticed. The treatment has been expectant, and the cases have done well. In my opinion there is generally too much interference in these cases. Erysipelas, both idiopathic and traumatic, has prevailed somewhat extensively, but the latter variety has come more under my observation. I have had three cases that terminated almost in exhausting suppuration. They all required operative proceedure. I gave a brief synopsis of those cases to the Society, but am now better prepared to give definite results.

The first case followed an injury received at the mechanical bakery. The hand and fore-arm were caught between iron rollers, and remained there three-fourths of an hour. The soft parts of the hand and fore-arm to near the elbow, save the muscular structures, were reduced to a pulp. In a few hours erysipelas, of an intense grade, followed in the wound and in the structures of the arm to the shoulder. Upon its subsidence, gangrene followed, involving all the lacerated tissue, and the sound integument to the shoulder. It became necessary for me to strip the hand and fore arm, both anteriorly and posteriorly, of all the mortified tissue, down to the muscles, tying arteries as I went. The symptoms in this case were typhoid, and the treatment from the beginning strongly supporting. I applied, locally, equal parts tinct. ferri muriatis, and strong tinct. iodine. Diet was strong beef-tea, well salted; iron and the sulphites, were also given. A part of our success may be ascribed to the latter remedy, as there become soon an amelioration in his symptoms after its use. The patient made a fair recovery, and has gone to New York to his parents.

The second case I reported went on to suppuration. An enormous quantity of matter was imprisoned beneath the integument and fascia of the foot and leg to the knee. A large incision set free an accumulation of most unhealthy pus—and he entered immediately upon convalescence.

The third and last case of traumatic erysipelas, I believe, we are now fully prepared to report. The soldier had received a shot from a pistol. The ball entered the upper third of the thigh, probably striking the great trochanter of the femur. Glancing from there I traced it several inches down the thigh beneath the fascia lata, and over the rectus muscle, when it was lost. No further attempt was made at finding it. Alarming erysipelatous inflammation followed speedily in the thigh, which eventuated in suppuration beneath the integument and fascia lata. The thigh become enormously swollen, and for days he would not allow me to open it, until the distension become so great that he could not endure it any longer. I made an incision four inches long, through the integument and fascia lata; an enormous quantity, it would seem a gallon, of most unhealthy pus escaped. Death, no doubt, would have followed in either of the cases mentioned before the matter would have found an external opening spontaneously. The idea is now fully established in my mind, that early, bold incisions are the only hope for our patients in such cases; for pus will hardly find its way through superficial and deep fascia in time to prevent a fatal degree of exhaustion.

We will not forget another pathological condition equally significant. There is a most poisonous agent to be absorbed into the blood. In suppuration from erysipelas cases, the pus, in consequence of its excessive alkalinity, saponifies and partly discolors the fatty tissue. The cellular tissue is broken down and escapes the wound with the adipose in the form of strings and shreds. A large handful escaped the wound a few hours after I made the incision in the last case mentioned. The direct operation in all these erysipelas cases is depressing, with marked prostration of the vital powers. The reabsorption of this poison causes early decomposition of the blood. The patient soon wears the livery of asthenia. Its presence is no less active in the rapid disintegration of the solid structures of the body, following more, in my experience, injuries, surgical operations, extreme privations, or excesses. The therapeutical indications in counteracting the effects of this zymotic agent, have been met by me with two classes of remedies. Antiseptics as the chlorides, and charcoal to operate from without. Strongly concentrated nutrient diet; tonics, especially of the ferruginous class, and the sulphite of lime to operate within. The symptoms in the last case were typhoid from the beginning. The blood had been rendered aplastic by previous dissipation. Traumatic delirium and coliquitive diarrhœa became two grave symptoms. Stimulants met the first indication, and the turpentine and laudanum emulsion the second. As soon as these symptoms were overcome, the muriated tinct. iron and the sulphites were given. The patient is convalescent.

We predicted in our last report, if changes were not soon wrought in the Sanitary condition of the river, and in our streets and alleys, there would be an augmentation of typhoid fever, as there was then but few cases, and there would be others of an adynamic and zymotic character. Our predictions have been verified, and well known to all the members of the Society. Fevers of the bilious remittent, intermittent, and typhoid type have prevailed; the latter to quite an extent this winter. Some cases have terminated fatally. They have all required, after suitable preparation of the system, quinine frequently, stimulants, and supporting treatment. There have been a number of cases of puerperal fever; some have been fatal. One physician told me he had been called to three cases in consultation, and all had died. Peritonitis and metro-peritonitis, I think, have been quite frequent, generally occurring after parturition. My treatment has been cathartics, anodynes, local synapisms to the abdomen, followed by fomentations of hops.

More cases of uterine disease have come under my observation than previously. The general symptoms are pain in the head, back, loins, and radiating down the thighs, general lassitude and debility. Upon examination, in the majority of cases, there is inflammation, and sometimes ulceration and granulation of the os and cervex uteri. In those cases there is often profuse leucorrhea, with disordered menstruation. My treatment has been local and constitutional. As an internal remedy, I use frequently the elixir calysaya ferratum, prepared by Sargent. As a local remedy I rely principally upon the nitrate of silver. I find the treatment much facilitated by daily vaginal injections of a saturated solution of alum. At the menstrual epoch, all local remedies are suspended, but continue the iron.

Two cases of retroversion of the uterus have come under my observation. The symptoms of each were nearly identical.

They complained of severe periodical pains in the lumbar region and small of the back. Anodynes had no effect excepting transitory in relieving the paroxysms. Upon a digital examination, I found the os patulous, and pointing to the symphisis pubis. The fundus of the uterus was retroverted upon the hollow of the sacrum, impinging much upon the rectum. The fundus was much hypertrophied, and unduly exalted in sensibility. My manipulations caused intense suffering. I reposited the organs, by pressure with the finger, within the rectum upon the fundus. This was all the interference necessary. Several cases of freezing have come under my observation, two of which terminated fatally; reaction in one was never established. In the other there was a hyper-reaction, delirium, and death. I believe nearly all the fatal cases could be traced to a too liberal use of some alcoholic stimulants. The fact, in my mind, is established that they all impair the calorific process; that the system can bear up longer under extreme low temperature without them than with them. Will the Society explain to me the pathology of freezing? Is its action chemical only? By destroying the endangium and all the essential properties, does the heart cease to respond to its stimuli? Are the springs of life frozen, or is its action primarily upon the the solid textures of the body?

Several cases of scurvy have come under my observation. They have all occurred among the poorly-clad and illy-fed of our city. My treatment has been fresh vegetable diet, lemon juice, and the sub-acid fruits, where circumstances would admit of their being procured. I ordered, before each meal, acid aromat. sulphuric; also warm ablutions to the skin.

Croup and convulsions have been frequent among children. I have had but little experience in the former. In the latter, have used chloroform, preceded by the hot bath, always with success.

There have been many cases of bronchitis, pneumonia, and pleuro-pneumonia, and congestion of the lungs. Several cases of the latter have terminated fatally. Tubercular consumption has abounded far more this year than last. There was an increase of twenty deaths this past January over last year's. I ascribe the cause to the exceeding low temperature, on the poorly-protected of our city. Much has been done in the milder form of this disease to alleviate and stay its onward march, but in the advanced tubercular variety, that healing cordial we have yet to find.

There have been a number of cases of diphtheria. They were all of the asthenic variety, having the well-marked aplastic exudation, and all required strongly supporting treatment.

More severe cases of surgery have come under my observation than heretofore. One interesting case of fracture of the lower jaw, treated successfully by silver wires passed around many of the teeth. The case made a good recovery by this method. The fracture was transverse at the symphisis, and at the ramus of the jaw. I have had a number of cases of compound comminuted fracture of the limbs. They were principally railroad accidents, and required amputation.

